# GaN nanorods grown on Si (111) substrates and exciton localization

**DOI:** 10.1186/1556-276X-6-81

**Published:** 2011-01-12

**Authors:** Young S Park, Mark J Holmes, Y Shon, Im Taek Yoon, Hyunsik Im, Robert A Taylor

**Affiliations:** 1 Department of Semiconductor Science, Dongguk University, Seoul, 100-715, South Korea; 2 Clarendon Laboratory, Department of Physics, University of Oxford, Parks Road, Oxford, OX1 3PU, UK; 3 Quantum Functional Semiconductor Research Center, Dongguk University, Seoul, 100-715, South Korea

## Abstract

We have investigated exciton localization in binary GaN nanorods using micro- and time-resolved photoluminescence measurements. The temperature dependence of the photoluminescence has been measured, and several phonon replicas have been observed at the lower energy side of the exciton bound to basal stacking faults (*I*_1_). By analyzing the Huang-Rhys parameters as a function of temperature, deduced from the phonon replica intensities, we have found that the excitons are strongly localized in the lower energy tails. The lifetimes of the *I*_1 _and *I*_2 _transitions were measured to be < 100 ps due to enhanced surface recombination.

PACS: 78.47.+p, 78.55.-m, 78.55.Cr, 78.66.-w, 78.66.Fd

## Introduction

The wide band gap semiconductor, GaN, and its heterojunction systems with AlGaN, has been intensively investigated during the past decade and has shown to be a very useful material for developing light emitting diodes, laser diodes, and high-power and high-temperature electronic devices [1,2]. It features a parabolic lowest conduction band with a band gap energy of approximately 3.4 eV. The separation between the conduction band and the nearest satellite valley is approximately 1.4 eV. Due to the properties of its constituents, it is also characterized by high-energy optical phonons (*ħ*ω_LO _≈ 92 meV).

Carrier localization in III-nitride materials caused by compositional fluctuations in ternary alloys leads to tailing of the energy bands that is observed in both absorption and photoluminescence (PL) spectra. Furthermore, it has been claimed that this localization gives rise to the high quantum efficiency commonly found in III-nitrides by preventing the carriers from reaching the dislocation level (which acts as many non-radiative recombination centers) [3,4]. These localizations are caused by alloy fluctuations in ternary semiconductors such as AlGaN [5,6] and InGaN [7,8] and in multi-quantum well structures such as AlGaN/GaN [9] and InGaN/GaN [10]. In binary systems such as GaN nanorods, clear identification of exciton localization with an appropriate analysis has rarely been reported. Exciton localization features have, however, been identified on basal stacking faults (BSFs) in *a*-plane epitaxial laterally overgrown GaN both experimentally [11] and in numerical calculations [12].

In this paper, we report on exciton localization within binary semiconductor GaN nanorods that are grown directly on Si(111) substrates. Time-integrated micro-photoluminescence and time-resolved photoluminescence (TRPL) experiments were carried out in order to study the optical properties of the GaN nanorods. The Huang-Rhys (H-R) parameters were calculated from the phonon replica intensities in order to understand exciton localization due to potential fluctuations.

## Experimental

### Sample preparation and characterization

**Figure 1:**
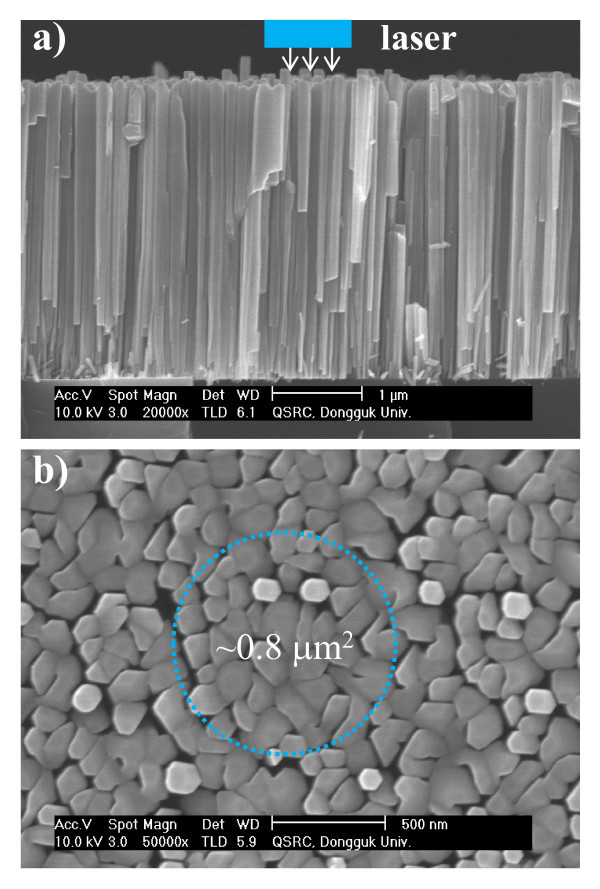
**SEM photographs of nanorods for top view (a) and side view (b)**. The light blue square-dotted circle shows the area principally excited by the laser in the micro-PL experiment.

The samples used in this study were grown on Si (111) substrates, without a buffer layer, by RF-plasma-assisted molecular beam epitaxy. The Ga source is a 7N5 pure metal in a conventional effusion cell. Nitrogen of 6N purity is further purified through a nitrogen purifier and then introduced into a plasma generator. A Si(111) substrate was degreased and then etched with diluted HF. The substrate was treated by thermal annealing at 1,000°C for 30 min. After deoxidation, the substrate temperature was lowered to 750°C for growth. The nanorod dimensions and density were determined by controlling the III/V ratio as well as growth time. The optical properties of GaN nanorods are determined mainly by the nanorods' dimensions, which in turn are strongly affected by the III/V ratio. More detailed growth conditions and techniques for GaN nanorods have been reported elsewhere [13-16]. Figure 1a, b shows high-resolution field emission scanning electron microscopy images at the cross-sectional and the plan view, respectively, for the bulk-like GaN nanorods. Two growth regimes are present in the sample, that is, a compact columnar growth from the Si substrate, and nanorods which protrude from the compact region. The compact region forms from the coalescence of nanorods. The average nanorod diameter is approximately 100 nm, and the average length is approximately 4 μm. More detailed information on the nanorods can be found in references [13,14].

### Photoluminescence measurements

A commercial micro-PL spectroscopy system (Renishaw Wotton-under-Edge, UK) was used for photoluminescence measurements. The excitation source was a He-Cd laser operating at 325 nm. This was focused to a spot size of approximately 0.8 μm^2 ^(marked with the dotted circle in Figure 1b) on the sample by a 36× reflecting objective positioned above a continuous-flow helium cryostat which housed the sample. The same objective was used to both focus the incident beam and to collect the resulting luminescence, which was subsequently directed to a spectrometer with a spectral resolution of approximately 700 μeV and a spatial resolution of 0.8 μm. The signal was detected using a charge-coupled device detector. For TRPL measurements, a frequency-tripled pulsed Ti:Al_2_O_3 _laser (100 fs at 76 MHz) was used to excite the samples at a wavelength of 266 nm. A commercial time-correlated single-photon counting system was used for detection.

## Results and discussion

**Figure 2:**
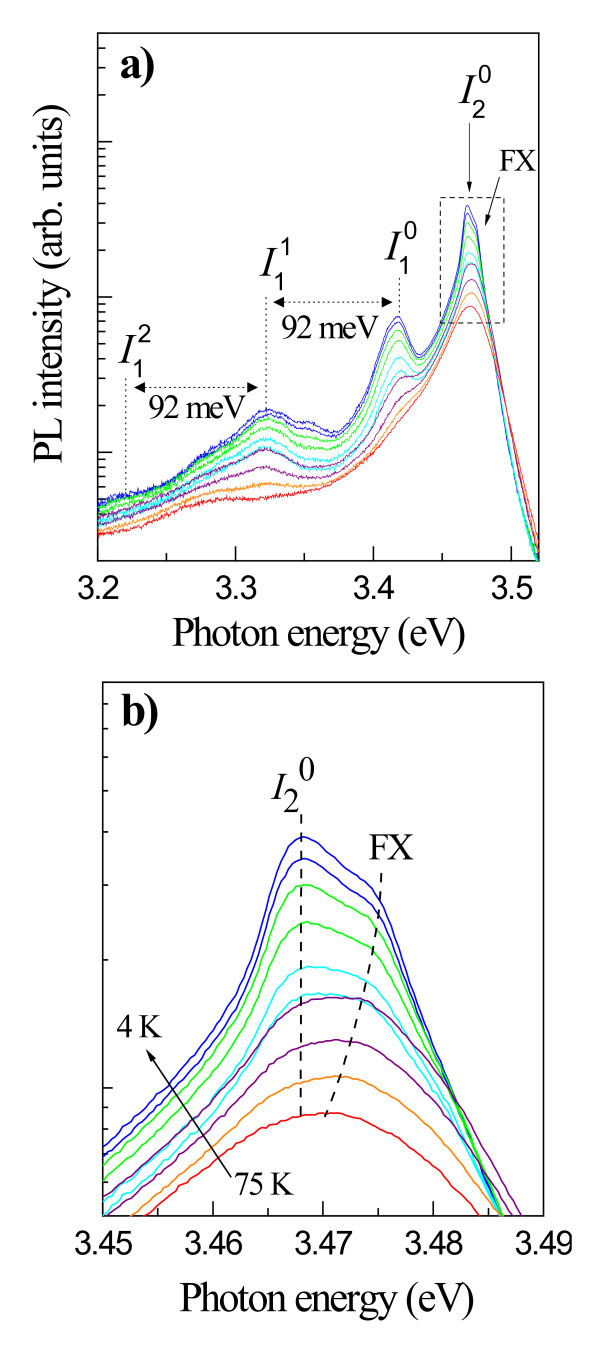
**Spectra of temperature-dependent PL**. **(a) **Temperature-dependent PL spectra at temperatures ranging from 4.2 K (higher intensity) to 75 K (lower intensity) and **(b) **in the extended scale.

The temperature dependence of the PL emission from the nanorods, measured at temperatures from 4.2 to 75 K, is presented in Figure 2a. Figure 2b shows a zoomed-in section of the spectra in which a strong excitonic emission, originating from both donor-bound excitons (*I*_2 _transition or D^0^X) and free excitons (FX) at energies of 3.468 and 3.476 eV, respectively, is observed. These peaks dominate the spectra at higher temperatures (note the log scale). The FX emission appears as a high-energy shoulder on the *I*_2 _peak, and we observe that it becomes red-shifted as the temperature increases, in line with the band gap energy dependence on temperature. At temperatures above 75 K, the *I*_2 _emission was deionized contributing to the FX emission. In contrast, the *I*_2 _emission energy appears to be temperature independent, which supports the assertion that it originates from a localized source. To the best of our knowledge, this localization effect has not previously been observed in GaN epilayers, and indeed, we only observe the effect in samples such as the one investigated here, which exhibits the coalescence of many nanorods.

In addition to these two peaks, there is a broad emission (full width at half maximum (FWHM), approximately 30 meV) at 3.417 eV, which has been labeled *I*_1_^0^. This has been attributed to both extended structural defects located at the bottom of the nanorods [17] and the recombination of excitons bound to the BSFs. The BSFs are produced during the initial phase of the growth and propagate perpendicularly to the *c*-axis from the substrate toward the surface of the sample [18,19]. Indeed, densely merged GaN nanorods on the surface (as shown in Figure 1) may result in the formation of the localized defects.

A series of satellite peaks at the low energy side of the aforementioned transitions, which is assigned to phonon replicas of the *I*_1_^0 ^emission, are also observed. The energy separation of each adjacent peak is approximately 92 meV, in good agreement with the longitudinal optical (LO) phonon energy in GaN. We denote the intensity of the nth phonon replica for *I*_1_^0 ^as *I*_1_*^n ^*(*n *= 0, 1, 2, *etc*), where *n *= 0 corresponds to the main emission line (non-replica). The intensity ratio of adjacent phonon replicas can be expressed as

(1) $$\begin{array}{cc} \frac{I^{n+1}}{I^{n}}\;\;=\;\;\frac{S_{n}}{n+1}\;, & n=0,\;\;1,\;\;2,\;3... \end{array}$$

where *S*, the H-R parameter, is defined as

(2) $$S\;\;=\;\;\sum_{q}\frac{{\left|V(q)\right|}^{2}}{E_{LO}}$$

Here, *E*_LO _is the LO phonon energy and *V*(*q*) is the matrix element for the interaction between the exciton and the phonon, with wave vector *q*. The H-R parameter is therefore a quantitative measure of the exciton-phonon coupling strength [20,21] and, by extension, a measure of the degree of localization (localized excitons have a stronger interaction with phonons as their wavefunctions contain large *q *components [22]).

**Figure 3:**
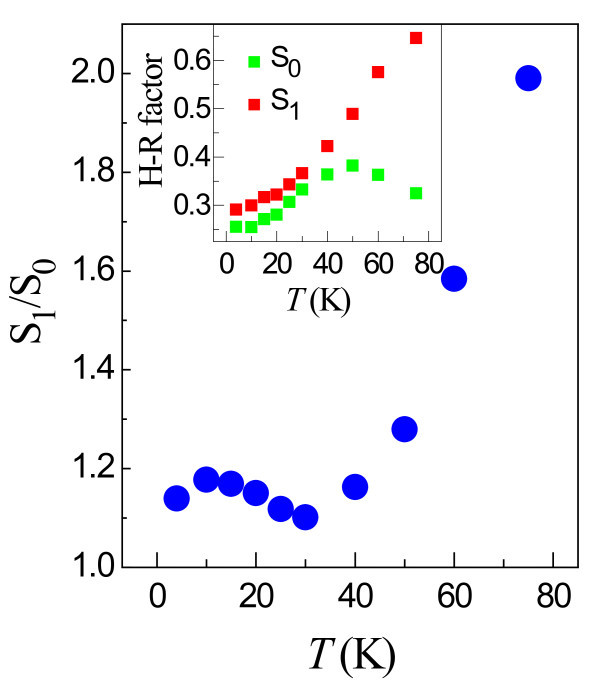
***S*_1_/*S*_0 _for the *I*1 transition as a function of temperature**. The inset shows the extracted value of the H-R factor for the *I*1 transition as a function of temperature.

The inset of the Figure 3 shows the extracted H-R parameters for the *I*_1 _transition as a function of temperature (*T*). The value of *S*_1 _at *T *= 4.2 K for the *I*_1 _is measured to be 0.29. It is a well-documented phenomenon that the value of *S*_0 _is always smaller than the H-R parameter measured between higher order satellite peaks [21] due to the fact that whilst all recombining excitons contribute to the zero order peak, only those that are deeply confined contribute meaningfully to the higher order satellites. The ratio of *S*_1_/*S*_0_, or the extent to which *S*_0 _is reduced by this effect, can therefore be used as a measure of the proportion of carriers that are localized [5,21]. Value of *S*_1_/*S*_0 _much in excess of unity represents a situation with few localized carriers. Figure 3 shows the temperature dependence of the ratio *S*_1_/*S*_0 _for the *I*_1 _transition. The value of *S*_1_/*S*_0 _initially decreases with increasing temperature (4.2 to 30 K) before rapidly increasing for temperatures above 30 K. Similar behavior has been observed previously in InGaN QWs [23] and Al*_x_*Ga_1 - *x*_N alloys [5] and is explained by exciton localization: Carriers that are strongly localized to deeper states at low temperature interact with the phonons but become delocalized by thermal energy with increasing temperature. The rise of *S*_1_/*S*_0 _for temperatures over 40 K roughly corresponds to, and is indeed caused by, the onset of a fall in *S*_0 _while *S*_1 _continues to rise. The continuing increase of *S*_1 _(and *S*_0 _for *T *< 50 K) with temperature is due to an increased population of phonons. The fall in *S*_0 _(*T *> 50 K) is most likely due to the delocalization of excitons at *I*_1_^0^, causing a reduction in the intensity at *I*_1_^1 ^(and indeed *I*_1_^2^). The emission intensity of *I*_1_^0^, however, is due to emission from all recombining excitons, so the thermal delocalization will have a less immediate effect on *I*_1_^0^resulting in a decrease of *S*_0 _and hence the increase of the ratio *S*_1_/*S*_0_.

**Figure 4:**
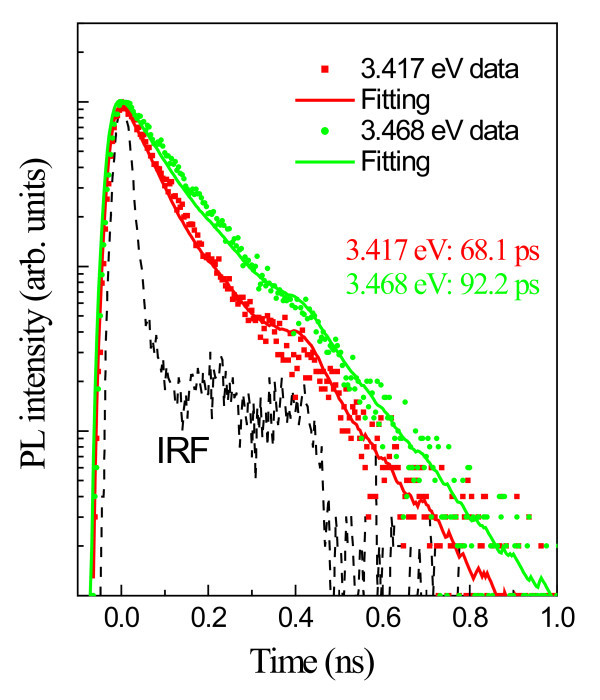
**Decay traces of time-resolved PL**. Time-resolved PL decay traces for the emissions at 3.417 eV (red squares) and at 3.468 eV (green circles). For reference purpose, the IRF is also shown (black dashed line). The decay time is deduced using a conventional fitting procedure (solid lines).

In order to further understand the carrier recombination dynamics of the excitons in GaN nanorods, we performed TRPL measurements on the sample at a range of photon energies. Two representative TRPL decay traces, taken at 10 K, along with the instrument response function (IRF), are presented in Figure 4. The shoulders in the IRF traces are due to electron reflections. This is a common problem in time-correlated photon counting system when relatively short time decays are involved. Software has been used to take into account this response function in the fitting procedure. The FWHM of the IRF is approximately 40 ps and was de-convoluted from the measured decays with commercial decay analysis software (PicoQuant Fluofit, PicoQuant GmbH, Berlin Germany). The mono-exponential lifetimes of the emission at 3.417 and 3.468 eV are calculated to be approximately 68.1 ps and approximately 92.2 ps, respectively. The decay rate is fast due to surface recombination on the nanorods, which is enhanced by the large surface to volume ratio exhibited by the columns. This is consistent with the results by Schlager *et al *[24]. The surface recombination lifetime, *τ*_s_, which is strongly dependent on the surface recombination velocity, *v*_s_, can be approximated to *d*/4*v*_s _for the case of a hexagonal column of diameter *d *[24,25]. We estimated the surface recombination velocity to be 27 × 10^3 ^cm.s^-1^, which is a little larger, but comparable, than that calculated by Schlager *et al*, owing to the faster decays observed in our case. It should be noted, however, that in the literature, there is little consistency in the lifetimes quoted for the donor-bound and acceptor-bound excitons. In fact, in GaN, the lifetimes range widely from a few tens to a few hundreds of picoseconds [26-31].

## Conclusions

In summary, we have investigated the exciton localization in bulk-like GaN nanorods by micro- and time-resolved photoluminescence measurements. In the temperature-dependent photoluminescence measurements, several phonon replicas at the lower energy side of the exciton bound to the BSFs (*I*_1_^0^) are observed. By analyzing the H-R parameter as a function of temperature deduced from the phonon replica intensities, we found that the excitons are strongly localized in the lower energy tails. For the *I*_1 _transition, the value of *S*_1_/*S*_0 _slightly decreases when the temperature increases from 4.2 to 30 K and then rapidly increases with further temperature increase, up to a value of 75 K. The PL decay times for the emissions at 3.468 and 3.417 eV were measured to be 92.2 and 68.1 ps, respectively. These fast decays are due to surface recombination, which is enhanced due to the large surface to volume ratio of the columns. It is finally concluded that exciton localizations in III-nitride materials can be observed not only in ternary alloys but also in binary semiconductors.

## Competing interests

The authors declare that they have no competing interests.

## Authors' contributions

YP carried out sample growth, performed PL measurements and drafted the manuscript. MH participated in PL measurements. YS participated in the structural analysis of the sample. IY participated in the growth of the sample. HI performed the data analysis and drafted the manuscript. RT participated in PL measurements and in the design of the study. All authors read and approved the final manuscript.

## References

[B1] Nakamura S Senoh M Nagahama S Iwasa N Yamada T Matsushita T Sugimoto Y Kiyoku H High-power, long-lifetime InGaN multi-quantum-well-structure laser diodes Jpn J Appl Phys 1997 36 Part 2 L1059

[B2] Ventury R Zhang NQ Keller S Mishra UK The impact of surface states on the DC and RF characteristics of AlGaN/GaN HFETs IEEE Trans Electron Devices 2001 48 560

[B3] Steude G Meyer BK Goldner A Hoffman A Bertram F Christen J Amano H Akasaki I Optical investigations of AlGaN on GaN epitaxial films Appl Phys Lett 1999 74 2456

[B4] Cho YH Gainer GH Lam JB Song JJ Yang W Jhe W Dynamics of anomalous optical transitions in Al*_x_*Ga_1*-x*_N alloys Phys Rev B 2000 61 7203

[B5] Lee KB Parbrook PJ Wang T Ranalli F Martin T Balmer RS Wallis DJ Optical investigation of exciton localization in Al*_x_*Ga_1-*x*_N J Appl Phys 2007 101 053513

[B6] Li J Nam KB Lin JY Jiang HX Optical and electrical properties of Al-rich AlGaN alloys Appl Phys Lett 2001 79 3245

[B7] Satake A Masumoto Y Takao M Tsunenori A Fumihiko N Masao I Localized exciton and its stimulated emission in surface mode from single-layer In*_x_*Ga_1*-x*_N Phys Rev B 1998 57 R2041

[B8] Pecharroman-Gallego R Edwards PR Martin RW Watson IM Investigations of phonon sidebands in InGaN/GaN multi-quantum well luminescence Mater Sci Eng B 2002 93 94

[B9] Sabooni M Esmaeili M Haratizadeh H Monemar B Paskov P Kamiyama S Iwaya M Amano H Akasaki I Exciton localization behaviour in different well width undoped GaN/Al_0.07_Ga_0.93_N nanostructures Opto-electro Rev 2007 15 163

[B10] Pecharroman-Gallego R Temperature and well number dependence of exciton localization in InGaN/GaN quantum wells Semicond Sci Technol 2007 22 1276

[B11] Cordir P Lefebvre P Levrat J Dussaigne A Ganiere JD Martin D Ristic J Zhu T Grandjean N Deveaud-Pledran B Exciton localization on basal stacking faults in *a*-plane epitaxial lateral overgrown GaN grown by hydride vapor phase epitaxy J Appl Phys 2009 105 43102

[B12] Cordir P Lefebvre P Ristic J Ganiere J-D Deveaud-Pledran B Electron localization by a donor in the vicinity of a basal stacking fault in GaN Phys Rev B 2009 80 153309

[B13] Park YS Lee SH Oh JE Park CM Kang TW Self-assembled GaN nanorods grown directly on (111) Si substrates: Dependence on growth conditions J Crystal Growth 2005 282 313

[B14] Park CM Park YS Im H Kang TW Optical properties of GaN nanorods grown by molecular-beam epitaxy; dependence on growth time Nanotechnology 2006 17 952 10.1088/0957-4484/17/4/019 21727365

[B15] Park YS Park CM Fu DJ Kang TW Oh JE Photoluminescence studies of GaN nanorods on Si (111) substrates grown by molecular-beam epitaxy Appl Phys Lett 2005 85 5718

[B16] Park YS Kang TW Taylor RA Abnormal photoluminescence properties of GaN nanorods grown on Si (111) by molecular-beam epitaxy Nanotechnology 2008 19 475402 10.1088/0957-4484/19/47/475402 21836271

[B17] Ristic J Calleja E Sanchez-Garcia MA Ulloa JM Sanchez-Paramo J Calleja JM Jahn U Trampert A Ploog KH Characterization of GaN quantum discs embedded in Al*_x_*Ga_1*-x*_N nanocolumns grown by molecular beam epitaxy Phys Rev B 2003 68 125303

[B18] Shreter G Guzzi M Melnik YV Vassilevski K Dmitiev VA Strunk HP Cathodoluminescence and transmission electron microscopy study of the influence of crystal defects on optical transitions in GaN Phys Status Solidi A 1999 171 325

[B19] Liu R Bell A Ponce FA Chen CQ Yang JW Khan MA Luminescence from stacking faults in gallium nitride Appl Phys Lett 2005 86 021908

[B20] Huang K Rhys A Theory of light absorption and non-radiative transitions in F-centres Proc R Soc A 1950 204 404

[B21] Mowbray DJ Kowalski OP Skolnick MS Hopkinson M David JPR Optical spectroscopy of AlGaInP based wide band gap quantum wells Superlatt Microstruct 1994 15 313

[B22] Brener I Olszakier M Cohen E Ehrenfreund E Azra R Pfeiffer L Particle localization and phonon sidebands in GaAs/Al*_x_*Ga_1-*x*_As multiple quantum wells Phys Rev B 1992 46 7927 10.1103/physrevb.46.7927 10002540

[B23] Paskov PP Holtz PO Monemar B Kamiyama S Iwaya M Amano H Akasaki I Phonon-assisted photoluminescence in InGaN/GaN multiple quantum wells Phys Status Solidi B 2002 234 755

[B24] Schlager JB Bertness KA Blanchard PT Robins LH Roshko A Sanford NA Steady-state and time-resolved photoluminescence from relaxed and strained GaN nanowires grown by catalyst-free molecular-beam epitaxy J Appl Phys 2008 103 124309

[B25] Zhao QX Yang LL Millander M Sernelius BE Holtz PO Surface recombination in ZnO nanorods grown by chemical bath deposition J Appl Phys 2008 104 073526

[B26] Eckey L Holst CJ Maxim P Hoffmann A Broser I Meyer BK Wetzel C Mohov EN Baranov PG Dynamics of bound-exciton luminescences from epitaxial GaN Appl Phys Lett 1996 68 415

[B27] Shan W Xie XC Song JJ Goldenberg B Time-resolved exciton luminescence in GaN grown by metalorganic chemical vapor deposition Appl Phys Lett 1995 67 2512

[B28] Godlewski M Bergman JP Monemar B Rossner U Barski A Time-resolved photoluminescence studies of GaN epilayers grown by gas source molecular beam epitaxy on an AlN buffer layer on (111) Si Appl Phys Lett 1999 69 2089

[B29] Im JS Moritz A Stevber F Harle V Scholz F Hangleiter A Radiative carrier lifetime, momentum matrix element, and hole effective mass in GaN Appl Phys Lett 1997 70 631

[B30] Harris JS Monemar B Amano H Akasaki I Exciton lifetimes in GaN and GaInN Appl Phys Lett 1995 67 840

[B31] Smith M Chen GD Li JZ Lin JY Jiang HX Salvador A Kim WK Aktas O Botchkarev A Morkoc H Excitonic recombination in GaN grown by molecular beam epitaxy Appl Phys Lett 1995 67 3387

